# Artificial Intelligence for the Diagnosis of Transthyretin Amyloid Cardiomyopathy: A Systematic Review of Machine Learning Application

**DOI:** 10.1155/crp/3289574

**Published:** 2026-09-17

**Authors:** Eyad Jamileh, Ahmed T. Elmewafy, Muhammad Abdullah Qadeer, Zuhaib Zulfiqar, Kiran Kang, Kaveh Hosseini, Ibrahim Antoun

**Affiliations:** ^1^ Furness General Hospital, University Hospitals of Morecambe Bay NHS Foundation Trust, Cumbria, England, UK; ^2^ School of Medicine, University of Sheffield, Sheffield, England, UK, sheffield.ac.uk; ^3^ School of Medicine, University of Cambridge, Cambridge, England, UK, cam.ac.uk; ^4^ Cardiovascular Diseases Research Institute, Tehran University of Medical Sciences, Tehran, Iran, tums.ac.ir; ^5^ Department of Cardiology, Copenhagen University Hospital-Herlev and Gentofte, Copenhagen, Denmark; ^6^ Center for Translational Cardiology and Pragmatic Randomized Trials, Department of Biomedical Sciences, Faculty of Health and Medical Sciences, University of Copenhagen, Copenhagen, Denmark, ku.dk; ^7^ Department of Cardiology, University Hospitals of Leicester NHS Trust, Glenfield Hospital, Leicester, UK, nhs.uk; ^8^ Division of Cardiovascular Sciences, Clinical Science Wing, University of Leicester, Glenfield Hospital, Leicester, UK, nhs.uk

**Keywords:** amyloidosis, artificial intelligence, ATTR-CM, cardiac amyloid, cardiac imaging

## Abstract

**Background:**

Transthyretin amyloid cardiomyopathy (ATTR‐CM) remains under‐recognised, and earlier identification is increasingly important with disease‐modifying therapy. Artificial intelligence (AI) applied to routine cardiovascular data may support screening, triage and diagnostic interpretation.

**Methods:**

MEDLINE, Embase, CINAHL, Web of Science and CENTRAL were searched from inception to August 2026 for studies evaluating AI/machine learning for ATTR‐CM detection, screening or classification, including broader cardiac‐amyloidosis models with separately extractable ATTR‐specific performance and models detecting imaging phenotypes relevant to ATTR‐CM screening. Risk of bias was assessed using an adapted QUADAS‐2 framework, with AI‐specific considerations informed by QUADAS‐AI development work; selected TRIPOD + AI and CLAIM domains were assessed descriptively. Owing to substantial clinical and methodological heterogeneity, meta‐analysis was not performed.

**Results:**

Twenty‐eight primary reports were included, representing > 120,000 report‐level participant entries, although overlapping cohorts precluded estimation of a unique‐patient total. Performance varied by modality and task. AUCs ranged approximately 0.55–0.97 for ECG‐containing models, 0.72–1.00 for echocardiography/POCUS and 0.70–0.85 for CT‐based single‐modality approaches; CMR differentiation of ATTR from AL achieved an AUC of 0.92. Nuclear‐imaging models often showed high discrimination, including external‐testing AUCs of 0.925–1.000, although several targeted tracer‐uptake phenotypes rather than definitive ATTR‐CM. Recurrent concerns included enriched populations, limited event counts, internal‐only testing, threshold optimisation and incomplete calibration reporting.

**Conclusions:**

AI shows promise for ATTR‐CM screening and automated interpretation, but reported discrimination should not be equated with real‐world clinical performance. Prospective multicentre validation, calibration at realistic prevalence, transparent reporting and workflow‐level evaluation are required before routine implementation.

## 1. Introduction

Transthyretin amyloid cardiomyopathy (ATTR‐CM) is caused by myocardial deposition of misfolded transthyretin and commonly presents with heart failure, increased ventricular wall thickness, arrhythmia or conduction disease. Recognition is frequently delayed because its phenotype overlaps with common cardiovascular disorders and because confirmatory testing is often undertaken only after clinical suspicion has been raised [1–3]. The importance of earlier diagnosis has increased with the availability of disease‐modifying therapy, which has shifted the clinical objective from recognition of advanced disease toward identification of patients earlier in the disease course [4].

Contemporary ATTR‐CM evaluation is multimodal. Electrocardiography and echocardiography provide widely available phenotypic clues; CMR can characterise myocardial infiltration and bone‐tracer scintigraphy can establish a nonbiopsy diagnosis of ATTR‐CM when grade 2 or 3 myocardial uptake is present in the appropriate clinical context and a monoclonal protein disorder has been excluded [2, 3]. CT and PET have additional or emerging roles in selected populations. Each modality, however, depends on the recognition of sometimes subtle patterns and is affected by referral pathways, disease prevalence, image quality and operator expertise.

AI and machine‐learning methods offer a potential way to identify multivariable patterns that may be difficult to recognise consistently by conventional interpretation. Previous reviews have considered AI in cardiac amyloidosis broadly, and modality‐specific systematic reviews and meta‐analyses have evaluated AI‐enhanced ECG models [5–11]. The rapidly expanding literature now includes large‐scale external validation, multimodal models, opportunistic POCUS, radiomics, synthetic‐data approaches and prospective implementation. The present review therefore focuses specifically on ATTR‐CM‐relevant diagnostic evidence across the full diagnostic pathway and emphasises methodological quality, validation design, prevalence, calibration, model transparency and cohort overlap rather than headline accuracy alone.

The objective was to systematically evaluate AI and machine‐learning models used for detection, screening, classification or clinically relevant imaging phenotypes of ATTR‐CM; to compare performance across modalities and stages of validation and to identify methodological requirements for clinical translation.

## 2. Methods

### 2.1. Protocol and Reporting

This systematic review was conducted in accordance with PRISMA 2020 [12] and was prospectively registered in PROSPERO (CRD420251230839) [13]. The complete PRISMA Checklist is provided in Supporting File 1. Risk of bias was assessed using an adapted QUADAS‐2 framework [14], with AI‐specific considerations informed by the QUADAS‐AI development framework [15]. AI‐specific reporting and validation characteristics were additionally mapped against selected domains from TRIPOD + AI [16] and, for imaging studies, CLAIM 2024 [17]. These reporting frameworks were used descriptively and were not converted into numerical quality scores.

### 2.2. Eligibility Criteria

Original peer‐reviewed studies were eligible if they evaluated an AI or machine‐learning model for ATTR‐CM detection, screening, classification, early prediction or diagnostic triage using ECG, echocardiography/POCUS, CT, CMR, PET, bone scintigraphy/SPECT/SPECT‐CT or a multimodal combination containing at least one of these diagnostic data sources. Broader cardiac‐amyloidosis models were eligible when ATTR‐specific diagnostic performance was separately extractable. Studies that automated imaging phenotypes directly used in ATTR‐CM screening pathways, such as high‐grade cardiac bone‐tracer uptake, were eligible even when the model endpoint was the imaging phenotype rather than final etiologic diagnosis, provided this distinction was explicitly retained in the synthesis. Studies restricted to AL amyloidosis, treatment monitoring without a diagnostic outcome, noncardiac ATTR manifestations, pure administrative‐claims/EHR case‐finding without a prespecified ECG or imaging component, reviews, editorials, conference abstracts without adequate data and reports without extractable quantitative diagnostic performance were excluded.

Goto et al. [18] was included because ATTR‐specific ECG and echocardiographic diagnostic performance was separately extractable. Duffy et al. [19] was excluded because cardiac amyloidosis was modelled as a composite aetiology of left ventricular hypertrophy without separately extractable ATTR‐CM diagnostic performance. The later international EchoNet‐LVH evaluation by Duffy et al. [20] was considered supportive evidence rather than an eligible ATTR‐CM diagnostic study because its primary diagnostic metrics were reported for cardiac amyloidosis overall and subtype‐specific diagnostic performance was not separately quantified.

### 2.3. Search Strategy

A comprehensive literature search was conducted in MEDLINE (via PubMed), Embase, CINAHL, Web of Science Core Collection and the Cochrane Central Register of Controlled Trials (CENTRAL) from database inception to August 2026. The search combined controlled vocabulary and free‐text terms relating to three principal concepts: (1) cardiac and transthyretin amyloidosis; (2) artificial intelligence, machine learning, deep learning, neural networks and radiomics and (3) relevant cardiovascular diagnostic modalities, including electrocardiography, echocardiography, ultrasound/POCUS, CT/CCTA, MRI/CMR, PET/PET‐CT, bone scintigraphy and SPECT/SPECT‐CT. Tracer‐specific terms included DPD, PYP, HMDP/HDP, florbetaben and florbetapir. No publication‐date restrictions were applied. The complete database‐specific search strategies and selected eligibility decisions for borderline reports are provided in Supporting File 2.

### 2.4. Study Selection and Data Extraction

Two reviewers independently screened titles and abstracts and subsequently assessed potentially eligible full texts against the prespecified eligibility criteria, with disagreements resolved by discussion. Data were extracted at study level for study design, setting, recruitment period, sample size, number and prevalence of ATTR‐CM or the stated imaging‐positive endpoint, input modality, model architecture, reference standard, partitioning strategy, internal and external testing, calibration, explainability, code/model availability and diagnostic‐performance metrics, including 95% confidence intervals where reported by the original studies. Development, tuning and testing datasets were recorded separately where reported. Cohort overlap was assessed using institution, recruitment period, investigators, model lineage and cohort descriptions; potentially overlapping reports were retained when they addressed distinct model‐development, validation or implementation questions but were not treated as independent patient evidence.

### 2.5. Risk of Bias and AI‐Methodology Appraisal

Risk‐of‐bias judgements were made across the four QUADAS‐2 domains of patient selection, index test/model conduct, reference standard and flow/timing, and were classified as low, high or unclear risk of bias. AI‐specific considerations were incorporated into the assessment, including data partitioning, model complexity relative to event counts, threshold optimisation, external testing and verification. Applicability concerns, where assessed, were classified separately as low, high or unclear. Attention was paid to case‐control enrichment, low event counts relative to model complexity, patient‐level versus image‐level splitting, reuse of tuning data for threshold selection, absence of independent external testing and incomplete verification. In parallel, selected TRIPOD + AI/CLAIM items were extracted to describe architecture rationale, data partitioning, internal testing, external testing, calibration, explainability and code/model availability. CLAIM terminology of internal testing and external testing is used where possible to avoid the ambiguity of the term validation.

### 2.6. Data Synthesis and Meta‐Analysis Feasibility

Formal meta‐analysis was not undertaken because the included reports did not estimate a sufficiently comparable diagnostic quantity across clinically and methodologically similar populations. Target labels varied among definitive ATTR‐CM, mixed cardiac amyloidosis with ATTR‐specific subgroup analysis and high‐grade tracer uptake suggestive of cardiac amyloidosis. Modalities and model inputs differed substantially, thresholds were study‐specific and frequently optimised internally and many reports provided AUCs without compatible 2 × 2 diagnostic data or variance estimates at a prespecified threshold. Furthermore, development, internal‐testing, external‐testing and implementation cohorts represented different stages of model evaluation, while several reports shared source populations or model lineages, limiting statistical independence. A single pooled AUC or bivariate sensitivity/specificity estimate was therefore considered clinically inappropriate. Results were synthesised narratively by modality and validation stage, with ranges of reported performance presented where appropriate.

## 3. Results

### 3.1. Study Selection and Evidence Base

**FIGURE 1:**
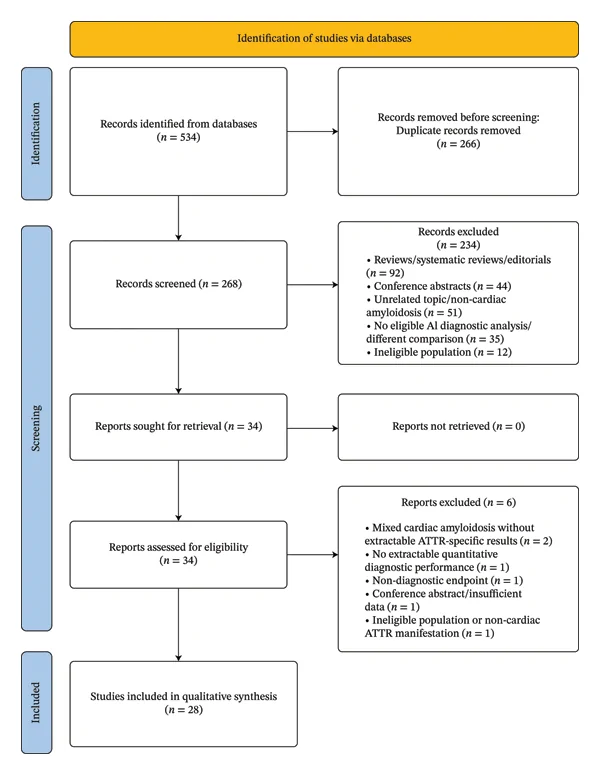
PRISMA flowchart.

The database search yielded 534 records. After removal of duplicates, 268 titles and abstracts were screened. Of these, 34 full‐text articles and conference abstracts were assessed for eligibility. Following a detailed review, 28 primary reports were identified for the current synthesis (Figure 1). The included reports spanned 2021–2026 and together represented more than 120,000 report‐level participant entries. This aggregate is intentionally not reported as a unique‐patient denominator because several publications shared institutions, model lineages or derivative cohorts and some studies reported multiple development and testing datasets (Tables 1 and 2).

**TABLE 1:** Characteristics and principal findings of included reports**.**

Study	Design/setting	*N* and target events	Modality/AI approach	Testing strategy	Main performance
Grogan et al. [21]	Retrospective matched case‐control; Mayo Clinic	4995; 2541 CA cases (mixed AL/ATTR; selected prevalence 50.9%)	12‐lead ECG; deep neural network	60/20/20 patient‐level train/internal‐test/holdout	AUC 0.91 (95% CI 0.90–0.93); PPV 0.86; 84% of holdout CA detected; signal preceded diagnosis in 59% by > 6 months
Harmon et al. [22]	Post‐development temporal testing; Mayo Clinic	7040; 440 CA, including 201 ATTRwt and 20 ATTRv	12‐lead ECG; previously developed AI‐ECG	Post‐development temporal testing using fixed prior threshold	Overall AUC 0.84 (95% CI NR); ATTRwt AUC 0.84 (0.81–0.87); ATTRv AUC 0.76 (0.63–0.89); sensitivity 64.3%; specificity 90.4% at prior threshold
Goto et al. [18]	Retrospective multicentre development and external testing	ECG cohorts > 9000; 324 ATTR cases across main ECG sites; separate echocardiography cohorts	ECG CNN and echocardiography deep learning	Held‐out derivation‐site testing plus external academic centres	ATTR‐specific ECG: BWH AUC 0.92 (95% CI 0.91–0.94), MGH 0.87 (0.84–0.90), UCSF 0.97 (0.95–0.98). ATTR‐specific echo: BWH 0.97 (0.96–0.98), MGH 0.94 (0.89–0.98), UCSF 1.00 (0.99–1.00), Northwestern 1.00 (1.00–1.00), Keio 0.96 (0.91–0.98)
Oikonomou et al., 2025 (POCUS) [23]	Retrospective multicentre; YNHHS + Mount Sinai	38,751 POCUS patients; YNHHS included 48 confirmed ATTR‐CM cases with prior/follow‐up POCUS	POCUS video CNN	Patient‐level development split; independent internal and external ED cohorts	ATTR‐CM AUROC 0.92 (95% CI NR) at YNHHS and 0.99 (95% CI NR) at Mount Sinai; 23/48 (47.9%) YNHHS cases screened positive a median 1.9 years before diagnosis
Oikonomou et al. (ECG/TTE) [24]	Retrospective two‐health‐system longitudinal study	1790; 286 ATTR‐CM (112/984 YNHHS; 174/806 HMH)	AI‐ECG and AI‐Echo CNNs	Held‐out testing and geographically distinct external cohort	AI‐Echo AUROC 0.93 (95% CI NR); AI‐ECG 0.91 (95% CI NR); probabilities diverged up to 3 years before nuclear diagnosis
Garmany et al. [25]	Retrospective clinical evaluation; tertiary referral cohort	598; 181 ATTR‐CA (30.3%); 28 AL excluded	Previously developed AI‐Echo and AI‐ECG	No retraining; predefined thresholds in scintigraphy‐referred cohort	AI‐Echo AUC 0.93 (0.91–0.95), sensitivity 86%, specificity 85%; AI‐ECG AUC 0.79 (0.76–0.83), sensitivity 80%, specificity 64%
Gonzalez‐Lopez et al. [26]	Retrospective single‐centre ATTR referral cohort	2901; 585 ATTR‐CM (20.2%); 20,754 ECGs	Time‐slicing CNN (Willem AI)	Patient‐level cross‐validation plus held‐out test; no external site	Test AUC 0.88 (0.85–0.91), sensitivity 80.7%, specificity 78.5%; ATTRv AUC 0.91 (95% CI NR), ATTRwt 0.88 (NR)
Hourmozdi et al. [27]	External comparative testing in heart‐failure population	3368; 176 confirmed ATTR‐CM (5.2%)	EchoNet‐LVH and EchoGo Amyloidosis; compared with claims RF and Mayo score	Independent external cohort; prespecified algorithms	EchoNet‐LVH AUC 0.88 (95% CI NR); EchoGo 0.92 (NR); Mayo score 0.79 (NR); claims RF 0.49 (NR); fairness audit performed
Slivnick et al. [28]	Multisite development with international external testing	Development 2612 (52% CA); external 2719 (597 CA; 22%)	Single apical four‐chamber video CNN ensemble	Five‐fold development; global external testing across 18 sites	External AUROC 0.93 (95% CI NR); sensitivity 85%; specificity 93% after 13% uncertain outputs; ATTRwt sensitivity 85%; ATTRv 86%; PYP‐referral subgroup AUROC 0.86 (NR)
Fraix et al. [29]	Retrospective derivation plus external multicentre testing	260 (111 ATTR‐CM; 42.7%) + external 454 (57% ATTR‐CM)	Decision‐tree ML using GLS, RVFWT, apical sparing and LV mass (GRAAL)	Internal cross‐validation; external multicentre cohort	Derivation AUC 0.90 (0.86–0.94); external AUC 0.83 (0.80–0.87); sensitivity 87%; specificity 65%
Mori et al. [30]	Four‐centre echocardiographic radiomics study	517; 261 ATTR‐CA (229 derivation + 32 external; selected case‐control)	Echocardiographic radiomics/logistic models	Leave‐one‐out CV in three centres; external fourth‐centre testing, *n* = 64	Internal AUC 0.97–0.99 (95% CI NR) for full‐ROI models; external AUC 0.95 (NR); accuracy 87%
Weberling et al. [31]	Multicentre CMR cohort from 56 institutions	400; 116 ATTR, 95 AL, 94 HCM, 95 healthy	Multiparametric CMR ML cascade	Random train/test plus sensitivity analysis using internal training/external referrals	ATTR vs AL AUC 0.92 (95% CI NR); HCM vs amyloidosis 0.99 (NR); healthy vs cardiac disease 1.00 (NR); ATTR‐vs‐AL fell to 0.88 (NR) using demographics + imaging only
Shiri et al. [32]	Prospective single‐centre severe‐AS cohort	263; 27 ATTR‐CM (10.3%)	Tabular ML across ECG, echo, 4D‐CT strain/radiomics and clinical data	70/30 split repeated across 100 random seeds; no external site	ECG < 0.68; CT radiomics 0.70–0.76; echo 0.79; CT strain 0.85; multimodal 0.84; 95% CIs NR for these estimates
Antonopoulos et al. [33]	Multicentre severe‐AS CT‐radiomics cohorts	589 across study arms; ATTR event counts varied by arm	CTA myocardial radiomics (AmyloidRS) + clinical variables	Training/validation cohorts by study arm	AmyloidRS c‐index 0.88 (95% CI NR) in training and 0.84 (NR) in validation; combined clinical + radiomics balanced accuracy 0.984
Nizam et al. [34]	Retrospective two‐centre study; severe‐AS/TAVR workflow	816; 127 ATTR‐CM (15.6%)	Multimodal CT + echo + ECG neural network	Separate TAVR workflow test cohort; authors note not true geographic external testing	Multimodal AUROC 0.85 (0.74–0.93); sensitivity 73.3%; specificity 82.9%; NPV 0.96; CT 0.70 (95% CI NR), echo 0.72 (NR), ECG 0.55 (NR); Brier score 0.081
Ramos‐Polo et al. [35]	Prospective single‐centre CRONOS‐ATTR	124; 95 ATTR‐CM (76.6%: 30 ATTRv, 65 ATTRwt)	XGBoost using clinical + ECG + echo AI‐derived features	Internal model development only	AUC 0.84 (95% CI NR); SHAP explanations; ECG + echo‐only model materially weaker
Santarelli et al. [36]	Prospective single‐centre PET study	47; 28 CA + 19 controls; subtype counts reported inconsistently across abstract/table	Early‐frame [18F]‐florbetaben PET CNN	Cross‐validation/internal testing only	ATTR sensitivity 0.935 (95% CI NR); specificity 0.897 (NR); accuracy 0.972 (NR); no external testing
Bargagna et al. [37]	Single‐centre PET neural architecture‐search study	47; 15 ATTR, 13 AL, 19 controls; same source cohort as Santarelli	[18F]‐florbetaben PET; NAS‐Net	Patient‐level splitting; repeated internal cross‐validation; no external site	ATTR sensitivity 93.3% ± 7.8%, specificity 78.0% ± 2.9%, accuracy 70.9% ± 3.7%; values reported as dispersion estimates rather than 95% CIs
Hong et al. [38]	Retrospective single‐centre multimodal nuclear study	50; 15 ATTR (30%), 12 AL, 23 non‐CA	Logistic ML using 11C‐PiB PET/CT + 99mTc‐DPD + clinical/imaging features	100‐fold stratified Monte Carlo CV; no external site	ML subtype AUC 0.94 (0.93–0.95), sensitivity 0.83, specificity 0.94; rationale‐based ATTR AUC 0.95 (95% CI NR); prognostic adjusted HR 2.43
Halme et al. [39]	Retrospective multicentre Finnish scintigraphy cohort	1334; 47 Perugini grade ≥ 2 (3.5%)	2D bone‐scintigraphy CNNs	Internal cross‐validation; no independent external testing	AUC > 0.88 (95% CI NR) for grade < 2 vs ≥ 2; AUC 0.94 (NR) for grade 3 vs lower grades
Delbarre et al. [40]	Retrospective two‐centre development/external‐testing study	4681; 383 Perugini ≥ 2 (281/3048 development; 102/1633 external)	Whole‐body bone‐scintigraphy CNN	Five‐fold internal CV at Henri Mondor; independent Lille external cohort	AUC 0.999 (95% CI NR) internally and externally; external sensitivity 96.1%; specificity 99.5%; estimated PPV falls substantially at low prevalence
Spielvogel et al. [41]	Retrospective international multicentre cross‐tracer study	16,241 patients/19,401 scans; 433 high‐grade uptake cases across cohorts	Whole‐body 99mTc scintigraphy deep learning	Austrian development; independent UK, Chinese and Italian testing	AUC 1.000 (95% CI NR) internally; external AUC 0.925–1.000 (CIs NR); AI outperformed readers in audit; adjusted mortality HR 1.44
Salimi et al. [42]	Retrospective multicentre multi‐tracer/multi‐scanner study	3747 across six datasets; labelled development and three external labelled cohorts plus 3215 unlabelled scans	Bone‐scintigraphy deep learning detection and severity scoring	External datasets using HDP, PYP and DPD	External detection AUC 0.956–0.999 (95% CIs NR); severity AUC 0.889–0.992 (CIs NR); Grad‐CAM/saliency used
Miller et al. [43]	Retrospective PYP SPECT/CT diagnostic/prognostic study	299; 83 ATTR‐CA (27.8%)	DL CT segmentation enabling automated SPECT quantification	Internal cohort only	CPA AUC 0.989 (0.974–1.000); VOI 0.988 (95% CI NR); TBR 0.979 (NR); quantitative measures predicted CV death/HF admission
Bhattaru et al. [44]	Retrospective two‐centre SPECT/CT study	170 (77 primary + 93 validation); 42/77 primary had abnormal myocardial uptake	U‐net automated myocardial segmentation; PYP/HDP SPECT/CT	Independent second‐centre testing	Diagnostic accuracy 100% (95% CI NR) in primary and 98% (NR) in validation; HBP ratio 3.1 ± 1.4 vs 1.4 ± 0.2
Haberl et al. [45]	Multicentre generative‐AI + downstream classification study	15,799 patients/16,823 scans; local classifier *n* = 181; 6448 patients across four external sites	Generative model + CNN uptake classifiers	Internal and cross‐tracer/cross‐scanner external testing	For CA‐indicative uptake: baseline internal AUC 0.900 (95% CI NR); external AUCs 0.968 and 0.978 (CIs NR) where positive cases were present; synthetic augmentation improved AUC by mean 5 ± 4 percentage points
Bati et al. [46]	Single‐centre curated PYP cohort	109; 62 Perugini 2–3 positive (56.9%), 47 negative	CNN + clinical metadata; Late Fusion and CMF‐Net	Train/validation/test split plus LOO and Monte Carlo CV; no external site	EfficientNet CMF‐Net accuracy 90.9% (95% CI NR); F1 91.4% (NR); ResNet‐50 late‐fusion sensitivity 94.9% (NR); enriched scan‐positivity endpoint
Jain et al. [47]	Model development/external testing plus prospective AI‐augmented clinical programme	ATTRACTnet: 799 internal + 422 external; implementation: 1471 AI‐positive, 256 eligible, 50 tested, 24 ATTR‐CM	Multimodal deep learning using ECG, echo, demographics and orthopaedic codes	Five‐fold internal CV, external cohort, then nonrandomised clinical implementation	AUC 0.85 (95% CI NR) internally and 0.82 (NR) externally; 24/50 (48%) tested AI‐positive patients had ATTR‐CM; 21/24 (88%) initiated treatment; 2.8‐fold diagnostic‐yield increase vs historical control

*Note:* AL, light‐chain amyloidosis; ATTR, transthyretin amyloidosis; ATTR‐CM/ATTR‐CA, transthyretin amyloid cardiomyopathy/cardiac amyloidosis; AUC/AUROC, area under the receiver‐operating‐characteristic curve; ECG, electrocardiography; HBP, heart‐to‐blood‐pool; HCM, hypertrophic cardiomyopathy; POCUS, point‐of‐care ultrasound; PYP, technetium‐99m pyrophosphate; TBR, target‐to‐background ratio; TTE, transthoracic echocardiography. Values in parentheses following AUC/AUROC estimates are 95% confidence intervals unless otherwise stated. NR indicates that a 95% confidence interval was not reported or was not available for the cited estimate. Values reported by the original studies as mean ± dispersion are retained in that form and are not presented as confidence intervals. Counts are study‐level entries and should not be summed as unique patients because of cohort/model overlap. Imaging‐uptake endpoints are identified as such and are not equated with definitive ATTR‐CM diagnosis.

Abbreviations: CA, cardiac amyloidosis; CMR, cardiovascular magnetic resonance; CT, computed tomography; HF, heart failure; NPV, negative predictive value; PPV, positive predictive value; VOI, volume of involvement.

**TABLE 2:** Potential cohort and model‐lineage overlap.

Reports	Assessment/implication
Santarelli et al. [36]/Bargagna et al. [37]	Same Pisa/Fondazione Toscana G. Monasterio [18F]‐florbetaben cohort; both use 47 subjects. Treat as derivative analyses, not independent participants
Spielvogel et al. [41]/Haberl et al. [45]	Both include Vienna bone‐scintigraphy data and overlapping external centres; Haberl explicitly reuses related cross‐centre infrastructure. Report separately but do not sum as independent evidence
Grogan et al. [21]/Harmon et al. [22]	Same Mayo AI‐ECG model lineage. Harmon is post‐development temporal evaluation of the previously developed model; cohorts are temporally distinct but algorithms are not independent
Grogan et al./Harmon et al. [21, 22]/Garmany et al. [25]	Mayo health‐system/model lineage. Garmany evaluates previously developed AI‐ECG and AI‐Echo in a scintigraphy‐referred cohort. Participant overlap with earlier Mayo reports should be regarded as possible unless ruled out by dates/identifiers
Slivnick et al. [28]/Garmany et al. [25]/Hourmozdi et al. [27]	Related Ultromics/EchoGo/EchoNet model families are evaluated across different cohorts. These are valuable external evaluations but not independent model‐development programmes
Oikonomou et al. (POCUS) [23]/Oikonomou et al. (ECG‐TTE) [24]	Both include Yale‐New Haven Health System data, but ascertainment pathways differ (ED POCUS vs nuclear amyloid referral). Potential participant overlap cannot be excluded
Shiri et al. [32]/Antonopoulos et al. [33]	No evidence of cohort overlap identified: studies were conducted in geographically distinct health systems (Bern vs Greece/Cyprus)
Hong et al. [38]/Spielvogel‐Haberl programme [41, 45]	Shared Vienna investigators and institutional data sources; direct participant overlap was not established but should be flagged as possible

The evidence bases included ECG models, echocardiography and POCUS, CMR, CT/radiomics, PET, bone scintigraphy/SPECT‐CT and multimodal systems. Sample sizes ranged from 47 participants in early PET studies to more than 16,000 patients in large‐scale scintigraphy studies and 38,751 patients in real‐world POCUS testing. ATTR‐CM prevalence varied from approximately 2%–5% in broad or prevalence‐targeted screening cohorts to more than 50% in deliberately enriched case‐control or imaging datasets, underscoring why predictive values and apparent classification accuracy cannot be compared directly across reports.

### 3.2. Electrocardiography‐Based AI

ECG‐based discrimination was heterogeneous rather than uniformly high. In the Mayo AI‐ECG study, Grogan et al. reported an AUC of 0.91 for the detection of mixed AL/ATTR cardiac amyloidosis [21]. In postdevelopmental temporal testing of the same model, Harmon et al. observed lower overall discrimination (AUC 0.84), with AUC 0.84 for ATTRwt and 0.76 for ATTRv, while specificity remained 90.4% at the previously defined threshold [22]. Goto et al. reported ATTR‐specific ECG AUCs ranging from 0.87 to 0.97 across academic‐centre cohorts [18]. Oikonomou et al. reported an AI‐ECG AUROC of 0.91 in a longitudinal ATTR‐CM nuclear‐testing cohort [24].

More recent dedicated ATTR studies reinforce the importance of the intended‐use population. Gonzalez‐Lopez et al. evaluated 20,754 ECGs from 2901 individuals, including 585 ATTR‐CM cases, and reported a held‐out AUC of 0.88 with 80.7% sensitivity and 78.5% specificity; discrimination was similar for ATTRv (0.91) and ATTRwt (0.88), although sensitivity was lower in asymptomatic and NYHA class I presentations [26]. In a high‐risk scintigraphy‐referred cohort, Garmany et al. reported an AUC of 0.79 for AI‐ECG, materially lower than the paired AI‐Echo model [25]. In the severe‐AS multimodal study by Nizam et al., ECG alone performed close to chance (AUROC 0.55) [34]. Across ECG‐containing analyses, reported AUCs therefore ranged from approximately 0.55 to 0.97, with the lower bounds emphasising the dependence of performance on phenotype, population and model lineage.

### 3.3. Echocardiography and Point‐of‐Care Ultrasound

Echocardiographic and POCUS models generally achieved moderate‐to‐high discrimination, but again with a broad range. Oikonomou et al. reported AI‐Echo AUROC 0.93 and demonstrated divergence of AI probabilities up to 3 years before nuclear diagnosis [24]. Their POCUS‐adapted CNN subsequently achieved AUROC 0.92 at Yale‐New Haven and 0.99 in the external Mount Sinai cohort; among 48 confirmed YNHHS ATTR‐CM cases with POCUS observations, 23 (47.9%) screened positive before confirmatory testing, a median 1.9 years earlier [23]. Garmany et al. reported AUC 0.93, sensitivity 86% and specificity 85% for a previously developed AI‐Echo model in 598 patients referred for scintigraphy [25]. Goto et al. additionally reported ATTR‐specific echocardiographic C‐statistics ranging from 0.94 to 1.00 across five academic‐centre cohorts, including 0.97 (95% CI 0.96–0.98) at BWH, 0.94 (0.89–0.98) at MGH, 1.00 (0.99–1.00) at UCSF, 1.00 (1.00–1.00) at Northwestern and 0.96 (0.91–0.98) at Keio [18].

Importantly, not all echocardiographic models approached these values. The simple GRAAL decision‐tree algorithm achieved AUC 0.90 in derivation but 0.83 in an external multicentre cohort, with 87% sensitivity and 65% specificity [29]. Mori et al. reported external AUC 0.95 for echocardiographic radiomics in a small fourth‐centre test cohort [30]. In external comparative testing in a heart‐failure population, Hourmozdi et al. found AUCs of 0.88 for EchoNet‐LVH and 0.92 for EchoGo Amyloidosis, compared with 0.79 for the Mayo clinical score and 0.49 for a claims‐based random‐forest model [27]. Slivnick et al. reported overall external AUROC 0.93 across 18 sites, but AUROC 0.86 in the clinically relevant PYP‐referral subgroup; subtype sensitivities were 85% for ATTRwt and 86% for ATTRv [28]. In Shiri et al., echocardiography alone produced AUC 0.79, and in Nizam et al., echo alone produced AUC 0.72 [32, 34]. Overall, echocardiography/POCUS AUCs ranged approximately 0.72–1.00.

### 3.4. Cardiovascular Magnetic Resonance‐Based AI

Weberling et al. evaluated 400 participants (116 ATTR, 95 AL, 94 HCM and 95 healthy volunteers) referred from 56 institutions [31]. The clinically most relevant subtype‐discrimination step, ATTR versus AL, achieved an AUC of 0.92. Higher AUCs of 0.99–1.00 applied to broader upstream tasks (HCM versus amyloidosis and healthy versus cardiac disease) and should not be interpreted as ATTR‐specific diagnostic accuracy. When the model was restricted to demographics and imaging data, ATTR‐versus‐AL discrimination fell to 0.88, illustrating the contribution of additional clinical information.

### 3.5. Computed Tomography, Radiomics and Multimodal AI

CT‐based performance was more modest than the highest nuclear‐imaging estimates. In 263 patients with severe AS, Shiri et al. reported AUC 0.70–0.76 for CT‐radiomic features, 0.85 for 4D‐CT strain and 0.84 for the multimodal model; importantly, multimodal integration did not outperform CT strain alone [32]. Antonopoulos et al. reported an AmyloidRS c‐index of 0.88 in training and 0.84 in validation, with very high balanced accuracy after combination with clinical variables [33]. Nizam et al. reported AUROC 0.70 for CT alone and 0.85 for the CT + echo + ECG multimodal model in an independent TAVR workflow test cohort; calibration was explicitly assessed (Brier score 0.081), although the authors cautioned that the affiliated‐centre workflow did not constitute true geographic external validation [34]. The prospective CRONOS‐ATTR study used XGBoost on clinical, ECG and echocardiographic data in 124 patients and achieved AUC 0.84, with SHAP explanations but no independent external test [35]. Thus, multimodal integration improved discrimination in some datasets but not consistently across studies.

### 3.6. PET and Molecular Imaging

The PET evidence remained small and methodologically heterogeneous. Santarelli et al. evaluated early‐frame [18F]‐florbetaben PET in 47 participants and reported ATTR sensitivity 0.935 and specificity 0.897 [36]. Bargagna et al. used neural architecture search on the same 47‐subject source cohort, with patient‐level data splitting; ATTR sensitivity was 93.3%, but specificity was lower at 78.0%, emphasising that the derivative reports should not be counted as independent patient evidence [37]. Hong et al. evaluated 50 patients with paired 11C‐PiB PET/CT and 99mTc‐DPD scintigraphy against biopsy/genetic data; the machine‐learning model achieved AUC 0.94 for subtype differentiation, and a rationale‐based ATTR classifier achieved AUC 0.95 [38]. These studies support feasibility but remain too small for firm conclusions regarding transportability.

### 3.7. Bone Scintigraphy and SPECT/CT

Bone scintigraphy and SPECT/CT studies included both direct ATTR‐CM classification and automated detection/quantification of the high‐grade cardiac uptake phenotype used in ATTR‐CM diagnostic pathways. Halme et al. reported AUC > 0.88 for discrimination of Perugini grades < 2 versus ≥ 2 and AUC 0.94 for grade 3 uptake, but only 47 of 1334 scans were grade ≥ 2 [39]. Delbarre et al. used a two‐centre design (3048 development/internal cross‐validation; 1633 independent external testing) and reported external AUC 0.999, sensitivity 96.1% and specificity 99.5% for abnormal cardiac uptake; the same paper demonstrated that positive predictive value would decrease markedly when the model was applied at low disease prevalence [40].

Spielvogel et al. provided the strongest external‐validation evidence for automated bone‐scan interpretation, including 16,241 patients and 19,401 scans from nine international centres. AUC was 1.000 in Austrian cross‐validation and 0.925–1.000 across independent UK, Chinese and Italian cohorts, with robustness assessed across tracers/scanners and AI predictions independently associated with mortality (adjusted HR 1.44) [41]. Salimi et al. similarly demonstrated multitracer/multiscanner external testing, with external detection AUCs approximately 0.956–0.999 and external severity‐scoring AUCs 0.889–0.992 [42]. Miller et al. used deep‐learning segmentation to automate PYP SPECT/CT quantification in 299 patients with 83 confirmed ATTR‐CA cases; cardiac pyrophosphate activity and volume of involvement achieved AUCs 0.989 and 0.988, respectively, and were associated with cardiovascular death or heart‐failure hospitalisation [43]. Bhattaru et al. reported 100% diagnostic accuracy in a 77‐patient primary cohort and 98% in a 93‐patient external cohort for an automated segmentation/quantification workflow [44].

Newer work has broadened the methodological focus. Haberl et al. used generative AI to augment a small training set and tested downstream uptake classifiers across four external sites; for the cardiac‐amyloidosis‐indicative task, reported AUCs reached 0.968 and 0.978 in external cohorts containing positive cases, while synthetic augmentation improved AUC by a mean 5 ± 4 percentage points [45]. Bati et al. combined PYP images with clinical metadata in 109 patients; EfficientNet CMF‐Net achieved 90.9% accuracy and 91.4% F1‐score, although the cohort was small, single‐centre and enriched for Perugini 2–3 positivity [46]. These studies reinforce the high technical potential of nuclear‐image AI as well as illustrate why high AUC/accuracy values based on uptake labels cannot be directly equated with end‐to‐end ATTR‐CM diagnosis.

### 3.8. Clinical Implementation and Prediagnostic Detection

Several reports moved beyond static discrimination. Oikonomou et al. demonstrated longitudinal divergence in ECG/echo model probabilities up to 3 years before nuclear diagnosis, while the POCUS study identified nearly half of confirmed cases with a positive screen a median 1.9 years before definitive diagnosis [23, 24]. Grogan et al. also reported AI‐ECG positivity more than 6 months before clinical diagnosis in 59% of detected cases [21]. Jain et al. evaluated a multimodal model (ATTRACTnet) using ECG waveforms, echocardiographic measures, demographics and orthopaedic diagnosis codes, followed by a nonrandomised clinical implementation programme. Model AUC was 0.85 internally and 0.82 externally; among 50 AI‐positive patients who underwent confirmatory testing, 24 (48%) had ATTR‐CM and 21/24 (88%) initiated treatment, with diagnostic yield 2.8‐fold higher than the historical comparator [47]. These findings are clinically important, but the verification of only a subset of eligible AI‐positive patients creates potential flow/verification bias and means implementation yield should not be interpreted as conventional test accuracy.

### 3.9. Methodological Quality, Reporting and Cohort Overlap

The adapted QUADAS‐2 assessment identified substantial methodological heterogeneity (Table 3). Recurrent high‐risk features were case‐control or deliberately enriched populations, very small positive‐event counts relative to model complexity, internal‐only testing, optimisation of thresholds or architectures within the available dataset, and use of imaging surrogates rather than complete etiologic ATTR‐CM confirmation. The index‐test/model domain was the most frequent source of concern. Stronger studies used patient‐level splitting, independent external sites, prespecified thresholds, explicit calibration or implementation‐level evaluation. Consequently, near‐perfect discrimination in selected datasets was not interpreted as direct evidence of equivalent performance in unselected clinical populations.

**TABLE 3:** Risk‐of‐bias assessment using an adapted QUADAS‐2 framework with AI‐specific considerations**.**

Study	Patient selection	Index test/AI	Reference standard	Flow/timing	Key rationale
Grogan et al. [21]	High	High	Low	Low	Matched case‐control design and internally optimised threshold; no geographic external testing
Harmon et al. [22]	Unclear	Low	Low	Low	Temporal post‐development testing improves transport assessment but remains within the same health system and uses matched sampling
Goto et al. [18]	High	Unclear	Unclear	Low	Large multisite testing, but case‐control enrichment and mixed cardiac‐amyloidosis target/reference standards limit applicability to ATTR‐CM
Oikonomou et al. (POCUS) [23]	Unclear	Unclear	Low	Low	Large real‐world external cohort; rare‐event label ascertainment and retrospective sampling remain potential sources of bias
Oikonomou et al. (ECG/TTE) [24]	Unclear	Unclear	Low	Low	External health‐system testing and longitudinal design, but the cohort was enriched through referral for nuclear testing
Garmany et al. [25]	Unclear	Low	Low	Low	Prespecified thresholds and absence of retraining are strengths; the high‐risk referral population limits generalisability
Gonzalez‐Lopez et al. [26]	High	High	Low	Low	Single‐centre enriched cohort, internal tuning and no external‐site testing despite held‐out patient testing
Hourmozdi et al. [27]	Unclear	Low	Low	Low	Independent heart‐failure testing and fairness assessment; prevalence was intentionally constructed through matching
Slivnick et al. [28]	High	Unclear	Unclear	High	Strong international external testing, but case‐control enrichment and uncertain/abstained outputs affecting approximately 13% of cases raise flow and applicability concerns
Fraix et al. [29]	Unclear	Low	Unclear	Low	External multicentre testing and an interpretable decision tree are strengths; retrospective suspected‐ATTR cohorts and uncertain grade‐1 classification remain concerns
Mori et al. [30]	High	Unclear	Low	Low	External‐centre testing was performed, but the study used case‐control enrichment and a small external sample
Weberling et al. [31]	High	Unclear	Low	Low	Multicentre recruitment was used, but selected phenotype groups and a modest sample for complex multistage modelling limit representativeness
Shiri et al. [32]	Low	High	Low	Low	Prospective consecutive cohort, but only 27 events were available and model selection was performed internally across a large feature space
Antonopoulos et al. [33]	Unclear	High	Low	Unclear	Radiomics development across study arms involved limited event counts and a highly selected severe‐aortic‐stenosis population
Nizam et al. [34]	Unclear	Unclear	Low	Low	Calibration was assessed and an independent workflow test was used, but affiliated centres and a small TAVR test cohort limit true external validation
Ramos‐Polo et al. [35]	High	High	Low	Low	Very small, high‐prevalence single‐centre cohort with internal‐only model development and evaluation
Santarelli et al. [36]	High	High	Low	Unclear	Very small cohort and internal image‐model evaluation; subtype counts were inconsistently reported
Bargagna et al. [37]	High	High	Low	Unclear	Derivative small PET cohort with internal architecture search and cross‐validation only, despite patient‐level splitting
Hong et al. [38]	High	High	Low	Low	Only 50 patients were included; evaluation relied on repeated internal cross‐validation without independent external testing
Halme et al. [39]	High	High	Unclear	Low	Only 47 positive scans were available; evaluation used internal cross‐validation and a visual uptake reference rather than definitive ATTR‐CM diagnosis
Delbarre et al. [40]	Unclear	Low	Unclear	Low	Strong independent second‐centre testing; the reference standard was expert Perugini grading rather than complete clinical ATTR‐CM confirmation
Spielvogel et al. [41]	Unclear	Low	Unclear	Low	Large multinational external testing and audit; the target was high‐grade cardiac uptake and two external cohorts were deliberately enriched
Salimi et al. [42]	Unclear	Unclear	Unclear	Low	Multicentre external testing and explainability were strengths, but labelled development/testing datasets were relatively small and the target was tracer uptake
Miller et al. [43]	Unclear	High	Low	Low	Clinically confirmed ATTR endpoint, but evaluation was retrospective, single‐centre and lacked independent external testing
Bhattaru et al. [44]	High	Unclear	Unclear	Low	Two‐centre testing was performed, but sample sizes were small and the principal endpoint was uptake/segmentation rather than end‐to‐end ATTR‐CM diagnosis
Haberl et al. [45]	Unclear	Unclear	Unclear	Low	Cross‐centre testing was a strength, but the task targeted abnormal tracer uptake and shared data lineage with previous scintigraphy studies
Bati et al. [46]	High	High	Unclear	Low	Small single‐centre enriched cohort with internally selected architecture/thresholds and a scan‐positivity surrogate endpoint
Jain et al. [47]	Unclear	Unclear	Low	High	External model testing and prospective implementation were strengths; only a subset of eligible AI‐positive patients underwent confirmatory testing, creating potential verification and flow bias

*Note:* Judgements reflect the diagnostic question used in this review. Risk of bias was assessed using an adapted QUADAS‐2 framework [14], with AI‐specific considerations informed by QUADAS‐AI development work [15]. Nuclear‐imaging studies targeting high‐grade cardiac tracer uptake were assessed against their stated index‐test target; applicability to end‐to‐end ATTR‐CM diagnosis is considered separately in the narrative synthesis.

The TRIPOD + AI/CLAIM mapping (Table 4) showed that external testing and explainability have become more common, but calibration and public model/code availability remain inconsistent. Explicit calibration was reported in only a minority of recent studies, notably Nizam et al. and Slivnick et al. Several imaging papers used Grad‐CAM, saliency maps, attention maps or SHAP; decision‐tree and radiomic models offered more intrinsically interpretable feature structures. Public source code was uncommon, although some studies, including Shiri and Bati, reported GitHub availability and Santarelli/Bargagna offered code on request.

**TABLE 4:** AI development, testing and reporting characteristics selected domains were mapped descriptively against TRIPOD + AI [16] and, where applicable, CLAIM 2024 [17].

Study	Architecture/rationale	Partitioning	Internal testing	External testing	Calibration	Explainability	Code/model availability
Grogan et al. [21]	Deep neural network; architecture described, justification limited	Patient‐level 60/20/20	Yes (internal holdout)	No geographic external	NR	NR	Not public/proprietary
Harmon et al. [22]	Evaluation of pretrained Grogan AI‐ECG; new architecture N/A	Post‐development temporal cohort; fixed prior threshold	N/A (evaluation study)	Temporal, same institution	NR	Subgroup performance audit	Proprietary
Goto et al. [18]	CNN‐based ECG and echo pipelines; architecture described	Patient‐level derivation/validation/test	Yes	Yes, multiple academic centres	NR	Limited; emphasis on downstream confirmatory pathway	Inference code reported; weights/data restricted
Oikonomou et al. (POCUS) [23]	View‐agnostic video CNN; design tailored to POCUS quality	Patient‐level training/validation/test	Yes	Yes (Mount Sinai)	NR	Grad‐CAM saliency maps	NR
Oikonomou et al. (ECG/TTE) [24]	EfficientNet‐based ECG and video echo models; prior validated architectures	Patient‐level development and held‐out testing	Yes	Yes (Houston Methodist)	Probability trajectories modelled; formal calibration NR	Longitudinal probability trajectories	Executable apps available on reasonable request
Garmany et al. [25]	Previously developed AI‐Echo/AI‐ECG; architecture N/A for evaluation	No retraining; prespecified thresholds	N/A	Clinical evaluation in independent referred cohort	Not recalibrated	Clinical utility analysis	Models proprietary
Gonzalez‐Lopez et al. [26]	Time‐slicing CNN; rationale linked to long ECG recordings	Patient‐level 5‐fold CV + test	Yes	No	NR	No formal explainability; acknowledged limitation	Willem platform/proprietary
Hourmozdi et al. [27]	External evaluation of four existing models	Independent HF cohort; no retraining	N/A	Yes	NR	Fairness/bias audit	Mixed; some proprietary
Slivnick et al. [28]	Ensemble of five 3D CNNs	Training + tuning + external cohorts; five‐fold training	Yes	Yes, 18 sites	Yes; calibrated probabilities/calibration curves	Uncertainty/abstention analysis; failure‐mode subgroup analyses	NR/proprietary
Fraix et al. [29]	Pruned decision tree (GRAAL); intentionally simple/interpretable	Repeated train/validation splitting + pruning	Yes	Yes, multicentre	NR	Intrinsic decision‐tree interpretability	NR
Mori et al. [30]	Logistic regression radiomics; feature selection tied to ROI‐dependence question	Leave‐one‐out CV	Yes	Yes, fourth centre	Machine‐specific image intensity calibration; probability calibration NR	Regression coefficients/feature ranking	NR
Weberling et al. [31]	36 candidate ML algorithms with grid search; 3‐stage cascade	70/30 split + 10‐fold CV model selection	Yes	Sensitivity analysis using internal‐only training/external referrals for testing	NR	Selected‐feature reporting	NR
Shiri et al. [32]	Multiple tabular ML classifiers and feature‐selection strategies	70/30 repeated over 100 seeds	Yes	No	NR	Feature‐selection outputs	Code/models publicly available on GitHub
Antonopoulos et al. [33]	Radiomics score + clinical model	Training and validation cohorts by study arm	Yes	Yes/held‐out validation cohorts	NR	Radiomic score/feature‐level interpretability	NR
Nizam et al. [34]	Modality‐specific encoders with multimodal fusion	Training cohort + separate TAVR workflow test	Yes	No true external site (authors explicitly caution)	Yes; Brier score 0.081 and reliability diagram	Shapley values + Grad‐CAM	NR
Ramos‐Polo et al. [35]	XGBoost selected for multimodal tabular prediction; grid‐search tuning	Single cohort internal development	Yes	No	NR	SHAP patient‐ and feature‐level explanations	NR; commercial component algorithms
Santarelli et al. [36]	Custom CNN (CAclassNet); architecture described	Cross‐validation/internal test	Yes	No	NR	NR	Custom code and anonymised images available on request
Bargagna et al. [37]	Evolutionary neural architecture search; explicit aim to automate architecture design	Patient‐level split; repeated internal splits	Yes	No	NR	Architecture visualisation	Code available on request
Hong et al. [38]	Logistic regression one‐vs‐rest + random survival forest	100‐fold stratified Monte Carlo CV	Yes	No	NR	SHAP feature importance	Data available on reasonable request; code NR
Halme et al. [39]	Custom Linear/Residual CNNs compared with standard architectures	Internal CV with augmentation and validation subset	Yes	No	NR	Maximum activation maps	NR
Delbarre et al. [40]	CNN classifier; model selected by F2 in 5‐fold CV	5‐fold CV then independent site test	Yes	Yes (Lille)	Predictive values modelled across prevalence; formal calibration NR	Activation heat maps	NR
Spielvogel et al. [41]	Deep‐learning cardiac‐uptake detector with medical algorithmic audit	Austrian CV then external sites	Yes	Yes, cross‐tracer/cross‐scanner multinational	NR	Attention maps + algorithmic audit	Model availability not clearly public
Salimi et al. [42]	Segmentation + ensemble classification networks	Internal development plus 3 labelled external datasets	Yes	Yes, multi‐tracer/multi‐scanner	NR	Grad‐CAM and saliency maps	Model/code sharing described; source data unavailable
Miller et al. [43]	Foundational DL CT segmentation used for quantitative SPECT metrics	Single‐cohort evaluation	Yes	No	NR	Interpretable quantitative CPA/VOI/TBR outputs	NR
Bhattaru et al. [44]	U‐net myocardial segmentation	Primary + independent second‐centre cohort	Yes	Yes	NR	Segmentation masks/quantitative HBP ratio	NR
Haberl et al. [45]	Generative model + downstream classifier; architecture motivated by data scarcity	Local training, internal + 4 external sites	Yes	Yes, cross‐tracer/cross‐scanner	NR	Reader study + downstream performance analysis	Representative synthetic data available; full pipeline availability limited
Bati et al. [46]	CNN image encoders + MLP; Late Fusion/CMF‐Net with attention	Patient‐level train/validation/test; LOO and 100‐iteration MCCV	Yes	No	NR	Attention‐based fusion; architecture ablations	Source code/model class publicly available on GitHub
Jain et al. [47]	ATTRACTnet multimodal deep learning; designed for clinical case enrichment	5‐fold internal CV + external model test + implementation cohort	Yes	Yes	NR	Clinical‐pathway outputs; feature contribution reporting limited	NR

*Note:* These are reporting descriptors, not a numerical quality score. NR = not reported/unclear in the publication; N/A = not applicable.

Potential participant or model‐lineage overlap was also clinically relevant (Table 2). Santarelli and Bargagna used the same 47‐subject florbetaben source cohort. Spielvogel and Haberl shared Vienna and related external scintigraphy infrastructure. Grogan and Harmon represent development and postdevelopment testing of the same Mayo AI‐ECG; Garmany subsequently evaluated previously developed AI models in a Mayo referral cohort. Related Ultromics/EchoGo/EchoNet model families appear across Slivnick, Garmany and Hourmozdi. These reports provide complementary evidence at different development stages but should not be treated as statistically independent studies or summed as unique participants.

## 4. Discussion

This systematic review demonstrates a broad and increasingly mature evidence base for AI‐assisted detection of ATTR‐CM. The available studies show a clear gradient of evidential maturity, with large externally validated cohorts supporting automated recognition of cardiac‐amyloidosis‐suggestive bone‐tracer uptake and echocardiographic phenotypes, while subtype‐classification, PET, CMR and smaller multimodal studies remain predominantly developmental. Overall, the evidence supports cautious optimism for AI‐assisted case enrichment, screening and diagnostic interpretation, but does not justify a general claim of uniformly high ATTR‐CM diagnostic accuracy across all modalities.

The largest nuclear‐imaging studies demonstrate impressive discrimination across scanners, tracers and countries. Spielvogel et al. is particularly important because its 16,241‐patient multinational dataset, independent cross‐tracer testing, reader comparison and prognostic analyses address several limitations of earlier single‐centre studies. However, the task was automated detection of high‐grade cardiac uptake suggestive of amyloidosis rather than the entire clinical algorithm required to distinguish ATTR from AL and other causes of uptake. This distinction matters: technical accuracy at reading a bone scan cannot replace the necessary assessment for a monoclonal protein disorder, SPECT confirmation where appropriate and clinical integration.

A consistent finding across modalities was that diagnostic performance depended strongly on the population and stage of model evaluation. Performance frequently decreased when models progressed from selected derivation cohorts to more clinically representative testing populations. For example, the Mayo AI‐ECG demonstrated an AUC of 0.91 in the development study by Grogan et al., compared with 0.84 during subsequent postdevelopment testing by Harmon et al. Similarly, the GRAAL algorithm achieved an AUC of 0.90 in derivation and 0.83 in external multicentre testing. In the comparative evaluation by Hourmozdi et al., two echocardiographic AI models retained good discrimination, whereas the claims‐based model performed close to chance. Nizam et al. similarly reported marked variation by input modality, with AUROC 0.55 for ECG alone compared with 0.85 for the multimodal model. Collectively, these findings emphasise the importance of validation setting and intended‐use population when interpreting reported discrimination and caution against relying on best‐performing estimates in isolation.

Prevalence is equally important. AUC describes ranking discrimination but does not determine the number of false positives that will arise in low‐prevalence screening. Several studies deliberately enriched cases or constructed case‐control datasets, while large consecutive scintigraphy cohorts contained only about 2% high‐grade uptake. Delbarre explicitly showed that positive predictive value could fall substantially when prevalence approached that expected in general populations. For deployment, calibration, PPV, decision‐curve/net‐benefit analysis, workflow capacity and downstream confirmatory testing are therefore at least as important as AUC.

Echocardiography and ECG are attractive because they are routinely acquired and can provide opportunistic signals before definitive testing. The longitudinal Oikonomou studies and real‐world POCUS data support the possibility that AI probabilities may become abnormal before conventional diagnosis. The Gonzalez‐Lopez data additionally show that dedicated ATTR‐CM AI‐ECG retains useful discrimination in early presentations, although sensitivity is lower among asymptomatic or NYHA I patients. These models should be conceptualised as triage or safety‐net tools that trigger confirmatory evaluation rather than stand‐alone etiologic diagnoses.

Multimodal integration is promising but not automatically superior. In Shiri, the multimodal model did not outperform CT strain alone. Nizam found meaningful improvement from integration, but the test cohort remained within affiliated academic workflows. CRONOS‐ATTR achieved interpretable multimodal prediction in a prospective setting but remains small and internally evaluated. Future multimodal research should therefore prioritise prospective external testing and calibration over simply increasing the number of input modalities.

The findings should also be considered in the context of the existing evidence syntheses. Previous systematic reviews have evaluated AI applications in cardiac amyloidosis broadly, while an ATTR‐CM‐specific systematic review and an AI‐ECG meta‐analysis have also been published [6–8, 10]. A 2026 meta‐analysis of machine‐learning diagnostic studies reported pooled ATTR‐CA sensitivity of 0.84, specificity of 0.85 and an SROC AUC of 0.91 [9]. The present review addresses a complementary question by examining AI across the multimodality ATTR‐CM diagnostic pathway, distinguishing models predicting definitive disease from those predicting imaging‐surrogate endpoints, evaluating model development and reporting characteristics and explicitly considering potentially overlapping cohorts and model lineages. Given the substantial clinical, methodological and target‐label heterogeneity identified, generation of a single additional pooled diagnostic estimate was considered unlikely to provide a clinically meaningful summary of the evidence.

Future studies should prospectively evaluate locked models in geographically, demographically and technically independent populations; report event prevalence and complete 2 × 2 performance at prespecified thresholds; assess calibration and clinical utility at realistic screening prevalence; use patient‐level data partitioning; publish model/version details and code where feasible and report subgroup performance by age, sex, race/ethnicity, ATTR genotype and important phenocopies. For multimodal AI, incremental value should be compared formally against simpler single‐modality models and established clinical scores. Implementation studies should measure diagnostic yield, false‐positive burden, confirmatory‐test utilisation, time to diagnosis, treatment initiation, patient outcomes and cost‐effectiveness.

### 4.1. Limitations

Several limitations should be acknowledged. First, the evidence base was dominated by retrospective studies and selected referral or case‐control populations, with relatively few prospective or implementation studies. Second, target definitions varied across studies: some models predicted aetiologically confirmed ATTR‐CM, others evaluated mixed cardiac amyloidosis with ATTR‐specific subgroup performance and several nuclear‐imaging studies predicted high‐grade cardiac tracer uptake rather than definitive ATTR‐CM. Third, several publications shared source cohorts, institutions or model lineages, preventing calculation of a defensible unique‐patient denominator and limiting statistical independence across reports. Fourth, calibration, use of prespecified thresholds, public availability of code or models and complete reporting of test‐set event counts were inconsistent. Finally, AI in ATTR‐CM is a rapidly evolving field, and continuing model development and external validation may alter the strength and applicability of the available evidence over time.

## 5. Conclusion

AI can identify ATTR‐CM‐relevant patterns across ECG, echocardiography/POCUS, CT, CMR, PET and nuclear scintigraphy, and several models have now demonstrated meaningful external or clinical‐workflow performance. Nevertheless, the evidence is not uniform: performance varies by task, population and stage of testing, while many studies remain susceptible to spectrum bias, low event counts, internal optimisation and incomplete calibration. AI is therefore best positioned at present as an adjunct for case enrichment, opportunistic screening and standardised interpretation, followed by established confirmatory pathways. Prospective multicentre validation and transparent reporting are required before routine adoption.

## Author Contributions

Eyad Jamileh and Ahmed T. Elmewafy collected and analysed data, contributed to drafting and revised the article. Muhammad Abdullah Qadeer, Zuhaib Zulfiqar and Kiran Kang collected and analysed data and contributed to drafting. Kaveh Hosseini and Ibrahim Antoun contributed to the design of the study and final approval.

## Funding

The authors received no financial support for the research, authorship and/or publication of this article.

## Ethics Statement

A statement of ethics is not applicable because this study is based exclusively on published literature.

## Conflicts of Interest

The authors declare no conflicts of interest.

## Supporting Information

Additional supporting information can be found online in the Supporting Information section.

## Supporting information


**Supporting Information** The supporting information accompanying this manuscript includes Supporting File 1, which contains the PRISMA 2020 checklist, and Supporting File 2, which details the comprehensive search strategy used across the databases.

## Data Availability

All data generated or analysed during this study are included in this article. Further enquiries can be directed to the corresponding author.
