# Supplementary material for: Artificial Intelligence for the Diagnosis of Transthyretin Amyloid Cardiomyopathy: A Systematic Review of Machine Learning Application
Source: Cardiol Res Pract. 2026 Sep 17;2026:3289574. doi: 10.1155/crp/3289574 (PMC13586670; doi:10.1155/crp/3289574)
Supplement: Supplementary file 1 [file CRP-2026-3289574-s001.zip › Supplementary_File_2_Search_Strategies_and_Supplementary_Material (1).docx]

Supplementary File 2- Electronic Search Strategies and Supplementary Methodological Material

Reference numbers correspond to those used in the main manuscript.

**Supplementary Methods S1. Electronic search strategy**

The search combined three concepts: (1) cardiac or transthyretin amyloidosis; (2) artificial intelligence, machine learning, deep learning, neural networks and radiomics; and (3) relevant diagnostic modalities, including electrocardiography, echocardiography/point-of-care ultrasound, CT, CMR, PET, bone scintigraphy and SPECT/SPECT-CT. Searches were conducted from database inception to the final search date shown below. No publication-date restriction was applied. Database-specific syntax is reproduced below.

MEDLINE (PubMed)

("Amyloid Cardiomyopathy"[Mesh] OR "Transthyretin Amyloidosis"[Mesh] OR "cardiac amyloidosis"[tiab] OR "amyloid cardiomyopathy"[tiab] OR "transthyretin amyloid cardiomyopathy"[tiab] OR "transthyretin cardiac amyloidosis"[tiab] OR "transthyretin amyloidosis"[tiab] OR "ATTR-CM"[tiab] OR "ATTR-CA"[tiab] OR ATTRwt[tiab] OR ATTRv[tiab] OR "wild-type transthyretin"[tiab] OR "hereditary transthyretin"[tiab]) AND ("Artificial Intelligence"[Mesh] OR "Machine Learning"[Mesh] OR "Deep Learning"[Mesh] OR "Neural Networks, Computer"[Mesh] OR "artificial intelligence"[tiab] OR "machine learning"[tiab] OR "deep learning"[tiab] OR "neural network*"[tiab] OR "convolutional neural network*"[tiab] OR CNN[tiab] OR "computer-aided"[tiab] OR "computer assisted"[tiab] OR algorithm*[tiab] OR radiomic*[tiab]) AND ("Electrocardiography"[Mesh] OR "Echocardiography"[Mesh] OR "Ultrasonography"[Mesh] OR "Tomography, X-Ray Computed"[Mesh] OR "Magnetic Resonance Imaging"[Mesh] OR "Positron-Emission Tomography"[Mesh] OR "Tomography, Emission-Computed, Single-Photon"[Mesh] OR "Radionuclide Imaging"[Mesh] OR electrocardiogra*[tiab] OR ECG[tiab] OR EKG[tiab] OR echocardiogra*[tiab] OR ultrasound[tiab] OR POCUS[tiab] OR "point-of-care ultrasound"[tiab] OR scintigraph*[tiab] OR "bone scan*"[tiab] OR "bone scintigraph*"[tiab] OR SPECT[tiab] OR "SPECT/CT"[tiab] OR PET[tiab] OR "PET/CT"[tiab] OR "computed tomography"[tiab] OR CT[tiab] OR CCTA[tiab] OR radiomic*[tiab] OR "magnetic resonance"[tiab] OR MRI[tiab] OR CMR[tiab] OR DPD[tiab] OR PYP[tiab] OR HMDP[tiab] OR HDP[tiab] OR pyrophosphate[tiab] OR florbetaben[tiab] OR florbetapir[tiab])

Embase

('amyloid cardiomyopathy'/exp OR 'transthyretin amyloidosis'/exp OR 'cardiac amyloidosis':ti,ab OR 'amyloid cardiomyopathy':ti,ab OR 'transthyretin amyloid cardiomyopathy':ti,ab OR 'transthyretin cardiac amyloidosis':ti,ab OR 'ATTR-CM':ti,ab OR 'ATTR-CA':ti,ab OR ATTRwt:ti,ab OR ATTRv:ti,ab) AND ('artificial intelligence'/exp OR 'machine learning'/exp OR 'deep learning'/exp OR 'neural network'/exp OR 'artificial intelligence':ti,ab OR 'machine learning':ti,ab OR 'deep learning':ti,ab OR 'neural network*':ti,ab OR 'convolutional neural network*':ti,ab OR algorithm*:ti,ab OR radiomic*:ti,ab) AND ('electrocardiography'/exp OR 'echocardiography'/exp OR 'ultrasonography'/exp OR 'computed tomography'/exp OR 'magnetic resonance imaging'/exp OR 'positron emission tomography'/exp OR 'single photon emission computed tomography'/exp OR 'scintigraphy'/exp OR ECG:ti,ab OR echocardiogra*:ti,ab OR ultrasound:ti,ab OR POCUS:ti,ab OR scintigraph*:ti,ab OR 'bone scan*':ti,ab OR SPECT:ti,ab OR 'SPECT/CT':ti,ab OR PET:ti,ab OR 'PET/CT':ti,ab OR 'computed tomography':ti,ab OR CT:ti,ab OR CCTA:ti,ab OR radiomic*:ti,ab OR 'magnetic resonance':ti,ab OR CMR:ti,ab OR MRI:ti,ab OR DPD:ti,ab OR PYP:ti,ab OR HMDP:ti,ab OR HDP:ti,ab OR florbetaben:ti,ab OR florbetapir:ti,ab)

CINAHL (EBSCOhost)

((MH "Amyloidosis+") OR TI ("cardiac amyloidosis" OR "amyloid cardiomyopathy" OR "transthyretin amyloid cardiomyopathy" OR "transthyretin cardiac amyloidosis" OR "transthyretin amyloidosis" OR "ATTR-CM" OR "ATTR-CA" OR ATTRwt OR ATTRv) OR AB ("cardiac amyloidosis" OR "amyloid cardiomyopathy" OR "transthyretin amyloid cardiomyopathy" OR "transthyretin cardiac amyloidosis" OR "transthyretin amyloidosis" OR "ATTR-CM" OR "ATTR-CA" OR ATTRwt OR ATTRv)) AND ((MH "Artificial Intelligence+") OR (MH "Machine Learning+") OR TI ("artificial intelligence" OR "machine learning" OR "deep learning" OR "neural network*" OR "convolutional neural network*" OR algorithm* OR radiomic*) OR AB ("artificial intelligence" OR "machine learning" OR "deep learning" OR "neural network*" OR "convolutional neural network*" OR algorithm* OR radiomic*)) AND ((MH "Electrocardiography+") OR (MH "Echocardiography+") OR (MH "Ultrasonography+") OR (MH "Tomography, X-Ray Computed+") OR (MH "Magnetic Resonance Imaging+") OR (MH "Positron Emission Tomography+") OR TI (electrocardiogra* OR ECG OR EKG OR echocardiogra* OR ultrasound OR POCUS OR "point-of-care ultrasound" OR scintigraph* OR "bone scan*" OR SPECT OR "SPECT/CT" OR PET OR "PET/CT" OR "computed tomography" OR CT OR CCTA OR radiomic* OR "magnetic resonance" OR MRI OR CMR OR DPD OR PYP OR HMDP OR HDP OR florbetaben OR florbetapir) OR AB (electrocardiogra* OR ECG OR EKG OR echocardiogra* OR ultrasound OR POCUS OR "point-of-care ultrasound" OR scintigraph* OR "bone scan*" OR SPECT OR "SPECT/CT" OR PET OR "PET/CT" OR "computed tomography" OR CT OR CCTA OR radiomic* OR "magnetic resonance" OR MRI OR CMR OR DPD OR PYP OR HMDP OR HDP OR florbetaben OR florbetapir))

Web of Science Core Collection

TS=(("cardiac amyloidosis" OR "amyloid cardiomyopathy" OR "transthyretin amyloid cardiomyopathy" OR "transthyretin cardiac amyloidosis" OR "transthyretin amyloidosis" OR "ATTR-CM" OR "ATTR-CA" OR ATTRwt OR ATTRv) AND ("artificial intelligence" OR "machine learning" OR "deep learning" OR "neural network*" OR "convolutional neural network*" OR algorithm* OR radiomic*) AND (electrocardiogra* OR ECG OR EKG OR echocardiogra* OR ultrasound OR POCUS OR "point-of-care ultrasound" OR scintigraph* OR "bone scan*" OR SPECT OR "SPECT/CT" OR PET OR "PET/CT" OR "computed tomography" OR CT OR CCTA OR radiomic* OR "magnetic resonance" OR MRI OR CMR OR DPD OR PYP OR HMDP OR HDP OR florbetaben OR florbetapir))

CENTRAL (Cochrane Library)

([mh Amyloidosis] OR [mh "Amyloid Cardiomyopathy"] OR [mh "Transthyretin Amyloidosis"] OR ("cardiac amyloidosis" OR "amyloid cardiomyopathy" OR "transthyretin amyloid cardiomyopathy" OR "transthyretin cardiac amyloidosis" OR "transthyretin amyloidosis" OR "ATTR-CM" OR "ATTR-CA" OR ATTRwt OR ATTRv):ti,ab,kw) AND ([mh "Artificial Intelligence"] OR [mh "Machine Learning"] OR ("artificial intelligence" OR "machine learning" OR "deep learning" OR "neural network*" OR "convolutional neural network*" OR algorithm* OR radiomic*):ti,ab,kw) AND ([mh Electrocardiography] OR [mh Echocardiography] OR [mh Ultrasonography] OR [mh "Tomography, X-Ray Computed"] OR [mh "Magnetic Resonance Imaging"] OR [mh "Positron-Emission Tomography"] OR [mh "Tomography, Emission-Computed, Single-Photon"] OR (electrocardiogra* OR ECG OR EKG OR echocardiogra* OR ultrasound OR POCUS OR "point-of-care ultrasound" OR scintigraph* OR "bone scan*" OR SPECT OR "SPECT/CT" OR PET OR "PET/CT" OR "computed tomography" OR CT OR CCTA OR radiomic* OR "magnetic resonance" OR MRI OR CMR OR DPD OR PYP OR HMDP OR HDP OR florbetaben OR florbetapir):ti,ab,kw)

**Supplementary Table S1. Selected eligibility decisions for borderline reports**

| Report | Decision | Reason |
| --- | --- | --- |
| Duffy et al., JAMA Cardiol 2022 [18] | Excluded | AI model phenotyped causes of left ventricular hypertrophy and reported cardiac amyloidosis as a composite category; ATTR-CM-specific diagnostic performance was not separately extractable. |
| Duffy et al., JACC Adv 2025 [19] | Supportive evidence; not included in primary synthesis | International EchoNet-LVH evaluation reported primary performance for cardiac amyloidosis overall; subtype-specific numeric diagnostic performance was not separately extractable. |
| Goto et al., Nat Commun 2021 [17] | Included | ATTR-specific ECG and echocardiographic diagnostic performance was separately extractable and met the prespecified eligibility criteria. |
